# Formulation of black pepper (*Piper nigrum* L.) essential oil nano‐emulsion *via* phase inversion temperature method

**DOI:** 10.1002/fsn3.1422

**Published:** 2020-02-21

**Authors:** Truong Dam Thai Vinh, Ly Thi Minh Hien, Dong Thi Anh Dao

**Affiliations:** ^1^ Faculty of Chemical Engineering HCMC University of Technology Viet Nam National University HoChiMinh City Ho Chi Minh City Vietnam; ^2^ Faculty of Biotechnology Open University Ho Chi Minh City Ho Chi Minh City Vietnam

**Keywords:** black pepper essential oil, emulsion, nano‐emulsion, phase inversion temperature, *Piper nigrum* L

## Abstract

Recent trends in preservation of processed foods involve the use of natural compounds, rather than chemically synthesized additives, to simultaneously confer antimicrobial properties and prevent fat oxidation. In this regard, black pepper essential oils, due to its diversity in biological activities, have been increasingly popular. The compounds are often used in relatively low amounts and in the form of nanoparticles to permit well blending into foods or uniform dispersion on the surface of fresh meat. The purpose of this study is to determine experimental parameters of a nano‐emulsion formation process from black pepper essential oil via the phase inversion temperature (PIT) technique. The study results showed that the system achieved the optimal nano‐emulsion under following condition: the ratio by weight of water: Tween‐80: oil = 86:9.7:4.3, the stirring speed of nano‐emulsions at 500 rpm for 45 min (heating at 75°C for 30 min and then rapidly cooling at 5°C for 15 min).

## Graphical abstract

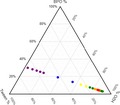

## 1 INTRODUCTION

Black pepper *(Piper nigrum* L.*,* Piperaceae family*)* is one of the most popular spices globally. Vietnam, one of the nations with largest black pepper sales, has been marked as a major exporter of this product with the production of black pepper of over 500 tons in 2018. The essential oils derived from black pepper find a myriad of applications in various fields, including manufacture of food and medicine, and therefore are commonly isolated from black pepper materials (Singletary, 2010). Black pepper essential oil appears as a liquid with the color varying from colorless to greenish and has a familiar peppery aroma. To date, approximately 46 compounds have been determined in black pepper oil (Butt et al., 2013) and many of which have been found to contribute to the strong antioxidant, anti‐inflammatory, and anti‐bacterial properties of the oil, suggesting the potential use of the oil as a preservative for food products (Butt et al., 2013; Singh, Marimuthu, Catalan, & deLampasona, 2004). However, due to poor solubility, absorbability into human body and dispersion of onto food of the black pepper essential oils are greatly limited. This calls for the development of a suitable method that is capable of producing particles of smaller size without compromising biological activities of the oils.

Emulsions were a relatively stable mixture of dissolved liquids, usually oil and water. Emulsion could be categorized into main types, namely oil‐in‐water emulsion (O/W) and a water‐in‐oil emulsion (W/O) (Esquerdo, Silva, Dotto, & Pinto, 2018; Jin, 2016) in which the former is commonly applicable in food products. In terms of physical state and stability, emulsions also consist of conventional emulsions (macro‐emulsion), nano‐emulsion, and micro‐emulsion (Jin et al., 2016). Among them, nano‐emulsion had a relatively small droplet size, ranging from 20 to 200 nm (Gupta, Eral, Hatton, & Doyle, 2016; Kumar & Mittal, 1999). Nano‐emulsion is dynamically stable and thermodynamically unstable. In recent years, nano‐emulsion technology has been extensively studied due to its high applicability in various industries such as polymer synthesis, food industry, pharmacology, or agriculture. Nano‐emulsions of essential oils, due to their high kinetic stability, low viscosity, and optical transparency, also interest the pharmaceutical and cosmetic industry (Izquierdo et al., 2004). Because nano‐particle size was relatively small, the system has a transparent, stable state against sedimentation or creaming (Forgiarini, Esquena, González, & Solans, 2001; Izquierdo et al., 2002). In addition, the manufacture of nano‐emulsion also requires a lower concentration of used surfactant in comparison with that of micro‐emulsion (Izquierdo et al., 2004). In food application, due to the poor solubility of vegetable essential oils, the use of nano‐essential oil emulsion may also facilitate the absorption of nutrients into the human body.

Recent interest in nano‐emulsion has shifted to optimization of the preparation process of nano‐emulsions containing essential oil, which showed improved bioactivities in comparison with the bare essential oil. Plant materials that were utilized in this process included *Salvia multicaulis* (Gharenaghadeh et al., 2017), clove/cinnamon (Zhang, Zhang, Fang, & Liu, 2017), oregano (Bedoya‐Serna, Dacanal, Fernandes, & Pinho, 2018), *Cleome viscosa* (Krishnamoorthy et al., 2018), and lemon myrtle/anise myrtle (Nirmal, Mereddy, Li, & Sultanbawa, 2018).

These notions have suggested the emulsification of black pepper essential oils as a pathway to enhance the use value and extend the applicability of this agricultural product. To fabricate nano‐emulsions, it is viable to employ high‐energy and low‐energy methods (Izquierdo et al., 2005). With high‐energy methods, it is necessary to maintain high mechanical energy exerting on the system using various kinds of instruments including ultrasound generators, rotor/stator type, and high‐pressure homogenizers (Ševčíková, Kašpárková, Vltavská, & Krejčí, 2012). On the contrary, the use of low‐energy emulsification methods requires low energy and the formation of nanoparticles is carried out mainly through either the phase inversion temperature (PIT) or the emulsion inversion point (EIP) technique (Anton, Benoit, & Saulnier, 2008). To date, all attempts to fabricate black pepper essential oil nano‐emulsion have been carried out via the high‐energy method only (Jiménez, Domínguez, Pascual‐Pineda, Azuara, & Beristain, 2018; Swathy, Mishra, Thomas, Mukherjee, & Chandrasekaran, 2018), leaving the low‐energy pathway unexplored with regard to this type of essential oil.

The PIT method is based on alterations in the hydration properties of nonionic surfactant head groups when the temperature changes. The head‐group is highly hydrated at relatively low temperatures but becomes progressively dehydrated as the temperature is increased (Anton & Vandamme, 2011; Roger, Cabane, & Olsson, 2009). This change in hydration alters the water solubility of the surfactant, as well as the optimum curvature of the surfactant monolayer (Figure 2). At low temperatures, the surfactants are more hydrophilic and favor the formation of oil‐in‐water systems, but at high temperatures they are more lipophilic and favor the formation of water‐in‐oil systems. At intermediate temperatures (around the PIT), the system tends to exist as a bicontinuous micro‐emulsion that is optically transparent (Rao & McClements, 2010). Therefore, we utilized the Phase inversion temperature method to formulate the essential oil nano‐emulsion (oil in water). The emulsifying effect is related to the hydrophilic–lipophilic balance (HLB) of emulsifiers (Lian, Peng, Shi, & Wang, 2019). HLB reflects the affinity of emulsifier to oil or water. The higher the HLB values are, the stronger the affinity to water is. As a rule of thumb, the phase in which the surfactant is more soluble would be the continuous phase. Therefore, hydrophilic surfactants (high HLB) tend to form oil in water emulsion, while hydrophobic surfactants tend to form water‐in‐oil emulsions.

In addition, Ahmad's studies (2013) have only shown that HLB values must be higher than 10 to be able to form oil in water nano emulsion. Tween – 80 had selected for oil phase because of its maximum emulsifying efficiency. Previous studies have experimented with some nonionic surfactants in O/W, including caprylocaproyl polyoxyl‐8‐glycerides (HLB = 14), polyoxyethylene (20) sorbitan monolaurate (HLB = 16.7), polyoxyethylene (20) sorbitan monooleate (HLB = 15), and polyoxyl‐15‐hydroxystearate (HLB = 15) (Ahmad, 2013).

In this study, we attempted to produce the black pepper essential oil nano‐emulsion via a low‐energy technique, the phase inversion temperature method (PIT). Some parameters of the process of preparing nano black pepper oil emulsion were investigated. The selected surfactant was Tween‐80 with an HLB index of 15, which is suitable for balancing nano‐emulsions.

## 2 MATERIALS AND METHODS

### 2.1 Materials

Distilled water (W) prepared at the laboratory, black pepper essential oil (BPEO) was supplied by An Phong Dak Nong company, Dak Song branch, village 10, Nam N'Jang commune, Dak Dong district, Dak Nong province. Black pepper essential oil was produced from Vietnamese black pepper by steam distillation. Emulsifier Tween‐80 (T‐80) was commercially obtained from Bio Basic (Canada) with HLB index = 15 suitable for domestic oil system (Americas, 1984). The used instruments consisted of a magnetic stirrer (VELP, Italy) and a viscometer.

### 2.2 Formulation of emulsions and nano‐emulsions

**Figure 1:**
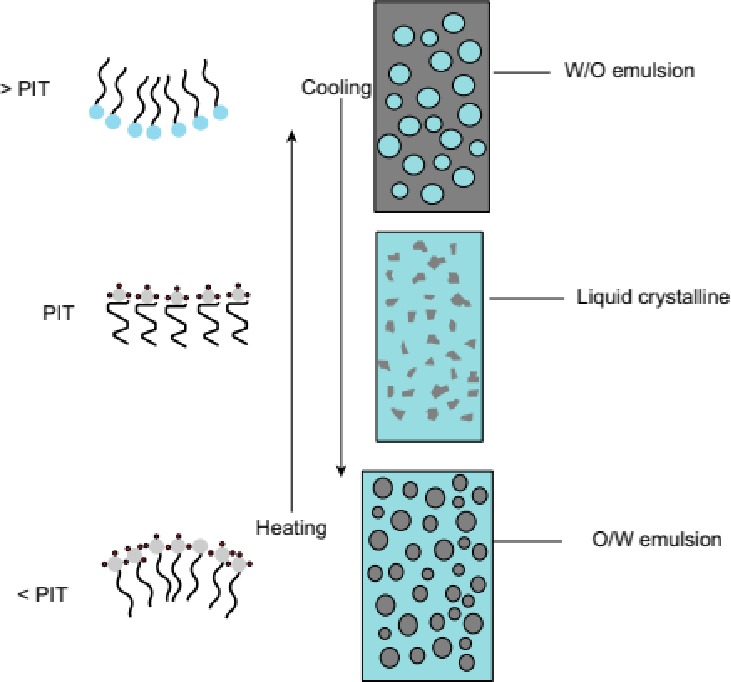
Structures of emulsifier layers in phase inversion temperature (PIT) method (Pathak, 2017)

**Figure 2:**
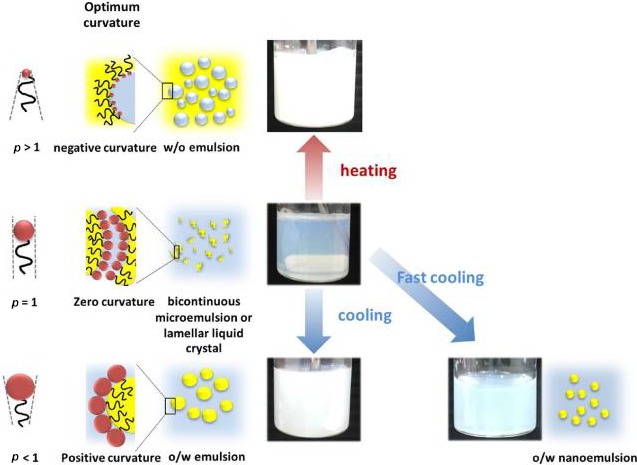
Schematic representation of the formation of nano‐emulsions by the PIT method (Jintapattanakit, 2018)

Black pepper essential oil nano‐emulsion was prepared by phase inversion temperature method consisting of two main phases (Figures 1 and 2). Structures of emulsifier layers in phase inversion temperature method (Figure 1) (Pathak, 2017).

Several researchers have introduced a packing factor (p) based on geometry of emulsifier molecular structure. Packing factor is represented as its hydrophilic and lipophilic behavior, with to be defined as (Chen, Xu, & Israelachvili, 1992; Waraho, McClements, & Decker, 2011):

(2.1) $$p=\frac{v}{la_{0}}$$

where:

- v is volume.
- l is length of hydrophobic tail.
- $a_{0}$ is cross‐sectional area of the hydrophilic head‐group.

The case of *p* < 1 (temperature is lower than PIT), oil‐in‐water emulsion is formed because the surfactant layer becomes convex resulting in hydrophilic behavior, whereas for *p* > 1, that is, at temperature higher than PIT, water‐in‐oil emulsion is constituted by the layer becomes concave resulting in lipophilic behavior. For *p* = 1 (at PIT), surfactant layer should be flat because of equal tendency of hydrophilic and lipophilic characteristic. The lamellar liquid crystalline systems should be constituted.

It is also to be noted that fine emulsions with small‐sized droplets are formed and surface tension decreases with increase in temperature. At PIT, droplets are often unstable as they try to coalesce and become macro‐emulsions. Maali and Mosavian (2013) have shown that a large amount of emulsifier is used to increase stability, droplets cannot coalesce immediately, and then some crystal structures of liquid are formed at PIT (Allouche, Tyrode, Sadtler, Choplin, & Salager, 2004). Thus, it is difficult to form emulsions near PIT because of instability. The stable and fine oil‐in‐water nano‐emulsions can be produced via a required cooling process; and required final nano‐emulsions must be preserved at a temperature far under from PIT (Liew, Nguyen, & Ngothai, 2010). Besides, if emulsions formed near PIT are cooled or heated immediately, the kinetically stability of emulsions can be constituted with small droplet size and narrow size distribution. Maali and Mosavian (2013) discussed about the transformation mechanism of oil‐in‐water emulsions. They also explained the difference of color and droplet size before and after inversion (Maali & Mosavian, 2013). Schematic representation of the formation of nano‐emulsions by the PIT method (Figure 2) (Jintapattanakit, 2018).

Phase inversion temperature can be determined by noting down the change in different properties of nano‐emulsions such as conductivity, viscosity, and turbidity (Rao & McClements, 2010). The conductivity of the system decreases and turbidity increases as nano‐emulsion switches over from oil‐in‐water to water‐in‐oil system. The temperature at which these properties drastically change is the PIT.

In the first phase, conventional oil‐in‐water emulsions were formed (under light underline heating. Briefly, the mixture containing Tween‐80 and black pepper essential oil at a specified T‐80: BPEO ratio was slowly added into distilled water. The ratio between BPEO and distilled water was maintained at 1:20. Afterward, the mixture underwent stirring at 500 rpm and heating at varying temperatures of 70°C; 75°C, and 80°C (Table 1) for 30 min. The total weight of the system was 30 g.

**Table 1:** The ratio of emulsifier to black pepper essential at different temperatures

Temperatures	SOR (w/w)	Denotation
70°C	2.25:1	A1
	2.5:1	A2
	2.75:1	A3
75°C	2.25:1	B1
	2.5:1	B2
	2.75:1	B3
80°C	2.25:1	C1
	2.5:1	C2
	2.75:1	C3

In the second phase, nano‐emulsions were formed by shrinking particles through rapid cooling. After stirring and heating for 30 min, heating was halted, followed by rapid cooling to 5 ÷ 10°C within 15 min.

### 2.3 Stability and phase separation of nano‐emulsions

Formed nano‐emulsions will be diluted 1,000 times in distilled water, then proceeded to absorbance measurement on Jasco V‐550 (UV–Vis) instrument after different periods including 1 day, 3 days, 1 week, and 1 month to determine the stability, durability, phase separation ability of the system (Jintapattanakit, 2018).

### 2.4 Analytical methods

Average diameters and polydispersity (PDI) indices of emulsions and nano‐emulsions were determined by the DLS method of Hosseinnia, Khaledabad, and Almasi (2017). The DLS method is based on vibrations corresponding with different intensities, produced by the Brown motion of particles along with the change in position in space. Dynamic light scattering (DLS) techniques use HORIBA SZ‐100 instrument. All measurements were carried out at 25°C. The plotting of particle size distribution was carried out directly by the HORIBA SZ‐100 instrument.

The GC‐MS (gas chromatography–mass spectrometry) method was used for determining the chemical components in the compound.

Viscosity was measured by a rotary viscometer, the Rion Viscotester VT‐04 (Japan), based on viscosity changes over time to determine the stability of nano‐emulsions.

Transmission electron microscopy (TEM) is a technique that studies microstructures of solid objects. The high‐energy electron beams from the instrument penetrate through specimens and lenses were used to create images, resulting in the magnification level of up to 400,000 times for most of materials and millions of times for atoms. Images were recorded with a digital camera on fluorescent screens or optical films.

## 3 RESULTS

### 3.1 The effect of the surfactant and essential oil ratio in nano‐emulsionss system

#### 3.1.1 Characterization of the nano‐emulsion fabricated under the heating temperature of 70°C

In this experiment, the essential oil nano‐emulsion was prepared with three levels of surfactant – oil ratios (SOR): 2.25, 2.5, and 2.75. After formation, the essential oil macro‐emulsions were heated up to 70°C before being rapidly cooled with cold water. The obtained nano‐emulsions were characterized for some parameters including viscosity, optical density (at 600 nm), average diameter size, and polydiversity index. This measurement wavelength was justified by other studies where nano‐emulsion characterization was evaluated at 600 nm (Akbas, Soyler, & Oztop, 2018; Sail, 2018; Salama, El‐Sayed, & Abd El‐Salam, 2016).

As the droplet diameter gets smaller than 80 nm, optical properties of the nano‐emulsion changed from being turbid to transparent (Roohinejad, 2018). This is similar to results of Sugumar et al. who fabricated the eucalyptus oil nano‐emulsion by simple mixing method at different of oil‐surfactant ratios (1:4, 1:3, 1:2 and 1:1). To be specific, at the ratio of 1:1, the milky white emulsion was obtained and products formulated at other ratios were transparent or translucent. It was also found in the same study that the transmittance value (measurement at 600 nm) was reduced but the average diameter rose when decreasing of oil‐surfactant ratio. In another study, Gharibzahedi and Mohammadnabi (2016) produced the stinging nettle essential oil nano‐emulsion by high‐pressure homogenization, showing the turbidity of the nano‐emulsion turbidity was positively related to the droplet diameters. These previous results imply that the emulsion systems with fine droplets had low turbidity.

**Figure 3:**
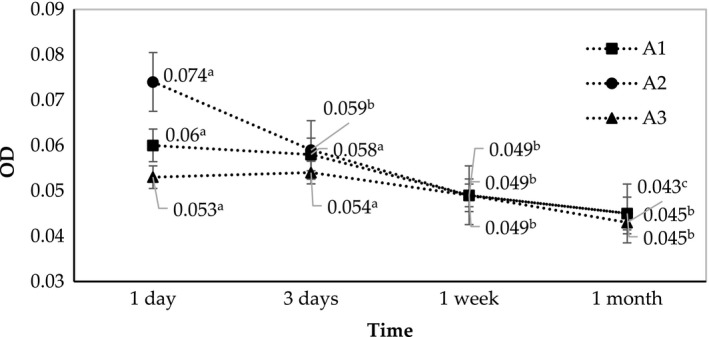
Changes in optical density of A1, A2, and A3 (fabricated at SOR 2.25, 2.5, and 2.75, respectively) during one month of storage

Obtained nano‐emulsions in our work were transparent with very low optical density (<0.1, measured at 600 nm) that persisted after one month of storage. These results affirmed the droplet size of our emulsion was below 80 nm. However, the OD values gradually decreased over a month, at room temperature. After one month, the optical density parameters of all treatments were almost identical. However, the SOR 2.5 and 2.75 samples (A2 and A3) seemed to be more stable because of the low initial OD and smaller drop in OD parameter (Figure 3).

Apart from optical property, viscosity was an important parameter that affects the formation of nano‐emulsion. Compared two low‐energy methods, which were heating and nonheating method, when fabricating the eucalyptus oil. The results showed that, in comparison with nonheated samples, the heated samples were transparent, and had lower viscosity and average droplet size.

**Figure 4:**
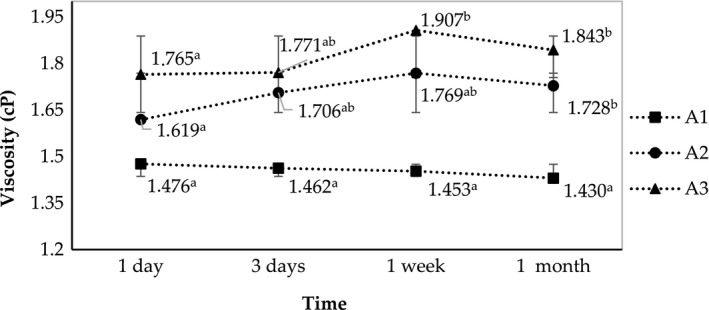
Changes in viscosity of A1, A2, and A3 (fabricated at SOR 2.25, 2.5, and 2.75, respectively)during one month of storage

Figure 4 shows that the viscosity was increasing with SOR (2.25, 2.5, and 2.75). The increasing of the viscosity determines the separation of the oil phase and prevents coagulation of the emulsion. However, very high viscosity could cause the system to get into the gel state, which is undesirable in nano‐emulsion production.

One‐month nano‐emulsion was measured for diameter size and the polydiversity index (PI) by Dynamic light scattering method (Table 2). The DLS result indicated that SOR of 2.5 and 2.75 are experimental conditions that produced emulsions with good average diameter size and low PdI, respectively, at 20.3 nm and 0.723 for SOR 2.5, and at 13.6 nm and 0.71 for SOR 2.75. Both of these SORs could be used to prepare nano‐emulsion at heating temperature of 70°C. The low physical parameters (the average droplet size <100 nm, the polydiversity index about 0.7 and the OD value <0.1) indicated the stability of the nano‐emulsions during 30 days storage at room temperature. However, since the PdI of these treatments was rather high (over 0.7), the heating temperature of 70°C was not the optimum condition for preparation of nano‐emulsion in our experiments.

**Table 2:** Average diameter size and polydiversity index of nano‐emulsion fabricated at 70°C

Samples	SOR (w/w)	Average diameter sizes (nm)	PdI
A1	2.25	14.0	3.396
A2	2.50	20.3	0.723
A3	2.75	13.6	0.710

#### 3.1.2 Characterization of the nano‐emulsion fabricated under the heating temperature of 75°C

The PIT method relies on changes in nonionic surfactant solubility with temperature. At the phase inversion temperature, the interfacial tension becomes extremely low and emulsification is promoted. By rapid cooling the system at the phase inversion temperature, kinetically stable emulsions with a very small diameter and narrow size distribution can be produced (Herrera, 2012). On the other hand, the phase inversion temperature of the emulsion depends on the composition. In this second experiment, SOR combinations were unchanged and the heating temperature before rapid cooling was elevated to 75°C.

**Figure 5:**
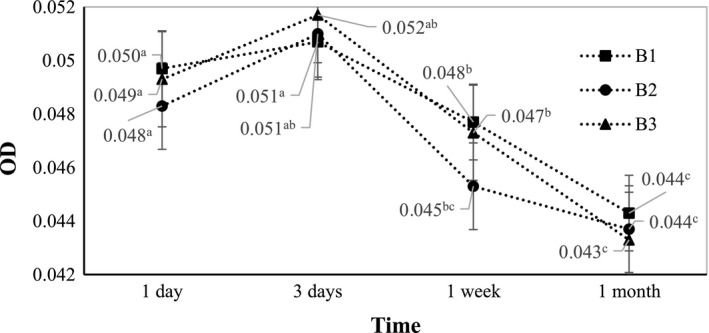
Changes in optical density of B1, B2, and B3 (fabricated at SOR 2.25, 2.5, and 2.75, respectively) during one month of storage

**Figure 6:**
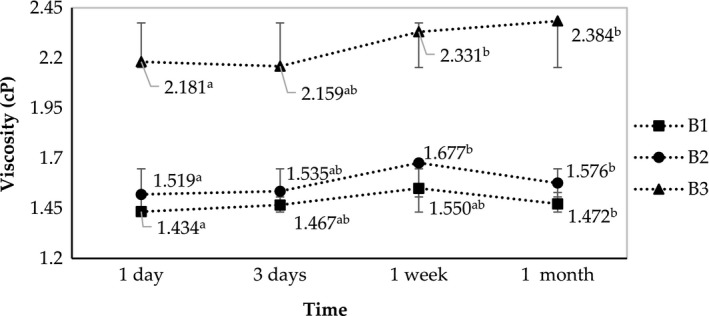
Changes in viscosity of B1, B2, and B3 (fabricated at SOR 2.25, 2.5, and 2.75, respectively) during one month of storage

The characteristic of the nano‐emulsions fabricated at 75°C heating was shown in Figure 5 and Figure 6. Although the viscosity of the B3 treatment was significantly higher than that of others, the measured optical densities of the three samples were almost identical. The OD values in this experiment during 30 days were all below 0.052, lower than those in the previous experiment where samples were heated at 70°C. This suggests that the temperature of 75°C is closer to the PIT and better facilitates the spontaneous emulsification. All three samples exhibited an increase in OD after 3 days then decreased to below 0.045 after one month of storage.

To select the optimum treatment, the samples were analyzed by DLS method. The results presented in Table 3 indicated that all of the samples were nano‐emulsions with very small average droplet size (below 50 nm). However, the polydiversity indices were inhomogeneous (in Table 3) and the optimum SOR at 75°C heating temperature was 2.25 (B1) (PdI 0.137). The sample B1 had an average diameter size of 17.9 nm and the narrow distribution in diameter size with the PdI of 0.137, which is indicative of high transparency and high physical stability.

**Table 3:** Average diameter size and polydiversity index of nano‐emulsion fabricated at 75°C

Samples	SOR (w/w)	Average diameter sizes (nm)	PdI
B1	2.25	17.9	0.137
B2	2.50	9.7	1.185
B3	2.75	43.6	0.969

#### 3.1.3 Characterization of the nano‐emulsion fabricated under the heating temperature of 80°C

**Figure 7:**
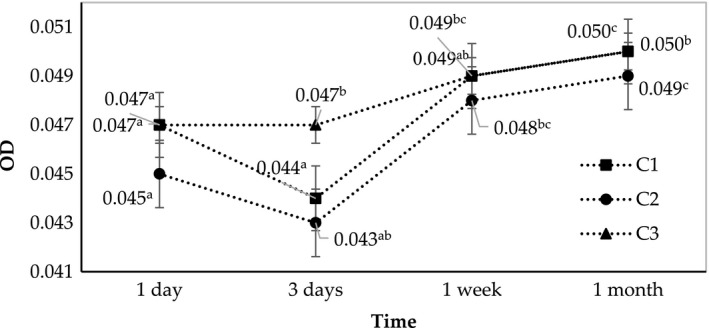
Changes in optical density of C1, C2, and C3 (fabricated at SOR 2.25, 2.5, and 2.75, respectively) during one month of storage

**Figure 8:**
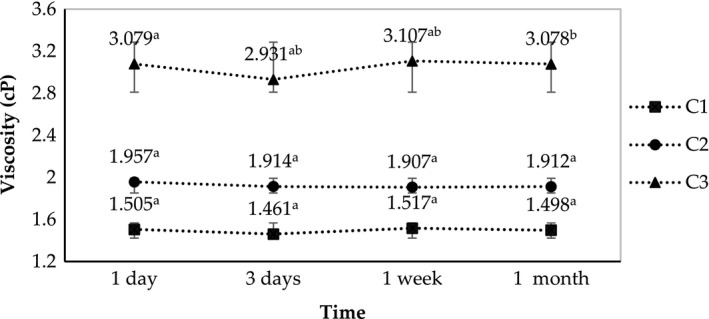
Changes in viscosity of C1, C2, and C3 (fabricated at SOR 2.25, 2.5, and 2.75, respectively) during one month of storage

In this last experiment, the heating temperature was elevated to 80°C. The higher heating temperature facilitates the stirring because of the reduced viscosity of the crude emulsion. However, excessively prolonged heating time may cause nano‐emulsion to insufficiently disperse due to the slower cooling rate. Similar to the last two experiments, the OD parameters of the three SORs showed minimal variations from each other during the one‐month storage (Figure 7). The SOR of 2.75 also achieved the highest viscosity in comparison with those of the remaining two samples (Figure 8).

The diameter size determination by DLS method in this experiment was different from the previous experiment (Table 4). The SOR 2.5 (C2) was the appropriate treatment at heating temperature 80°C, while the SOR 2.25 (B1) was the optimal one at 75°C heating treatment. This is consistent with previous studies demonstrating that that the emulsion PIT depended on the surfactant concentration (Jintapattanakit, 2018; Ontiveros et al., 2014; Ren et al., 2019). For example, Ontiveros et al. who investigated the effect of surfactant concentration on the PIT of the emulsion showed that, when the nonionic emulsifier HLB (hydrophilic–lipophilic balance) was higher than 10, the PIT of the emulsion was often positively proportional with the surfactant concentration (Ontiveros et al., 2014). This conclusion was in line with our experiment, in which the rise in the surfactant (Tween‐80) concentration from SOR 2.5 to SOR 2.75 necessitated the elevation in heating temperature to achieve the optimum nano‐emulsion.

**Table 4:** Average diameter size and polydiversity index of nano‐emulsion fabricated at 80°C

Samples	SOR (w/w)	Average diameter sizes (nm)	PdI
C1	2.25	10.2	1.189
C2	2.50	16.3	0.298
C3	2.75	12.3	10.506

Generally, both of the B1 and C2 treatments were the nano‐emulsion with desirable properties including low average droplet size (respectively, at 17.9 and 16.3 nm) and low polydiversity index (respectively, at 0.137 and 0.298). On the other hand, since the evaporation of black pepper essential oil is effortless at high temperature, the 75°C was the optimal temperature for heating and the B1 treatment was the optimum combination.

### 3.2 Characterization of the Nano‐emulsion Fabricated at the Optimum Treatment (B1)

**Figure 9:**
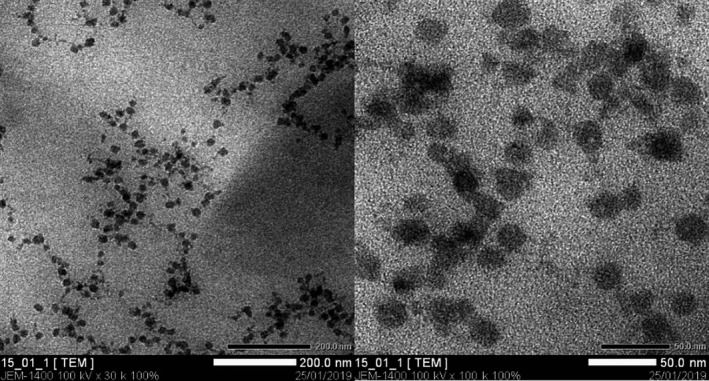
TEM images of the nano‐emulsion with SOR 2.25 and heating temperature at 75°C (B1)

The optimum nano‐emulsion was subject to TEM method for analysis of diameter distribution. The results are shown in Figure 9.

As shown in the TEM image (Figure 9), fabricated black pepper essential oil nano‐emulsion droplets were spherical in shape with the maximum mean diameter of approximately 30 nm. This result was confirmed by dynamic light scattering method, which measures the droplet size. Moreover, the emulsion was homogenous without particle coagulation indicating that the nano‐emulsion could maintain stability after 30 days of storage.

Figure 9 illustrates the size distribution of the nano‐droplets. Approximately, the size of the nano‐emulsion with SOR 2.5 followed a bell‐shaped distribution and ranged from 8 to about 100 nm, half of which were sized at lower than 50 nm in diameter. Mean diameter and PdI of droplets was 17.9 nm and 0.137, respectively, which is indicative of a nano‐emulsion system of high homogeneity. These results were consistent with TEM images (Figure 9) where nano‐droplets of black pepper essential oils were uniformly depicted at the size of lower than 50 nm. This figure is lower than the mean diameter of black pepper essential oil nano‐emulsion fabricated in two previous attempts via high‐energy methods (Jiménez et al., 2018; Swathy et al., 2018) in which the produced nano‐emulsions averaged below 100 nm and could effectively control bacterial infections in aquaculture and foods.

In comparison with the results of Su and Zhong (2016) who employed PIT to fabricate nano‐emulsions of lemon oil with Tween 20 emulsifier and measure the particle size after 15 days of storage, the presented results achieved the particle size at a much smaller diameter (17.9 vs. 100 nm).

**Figure 10:**
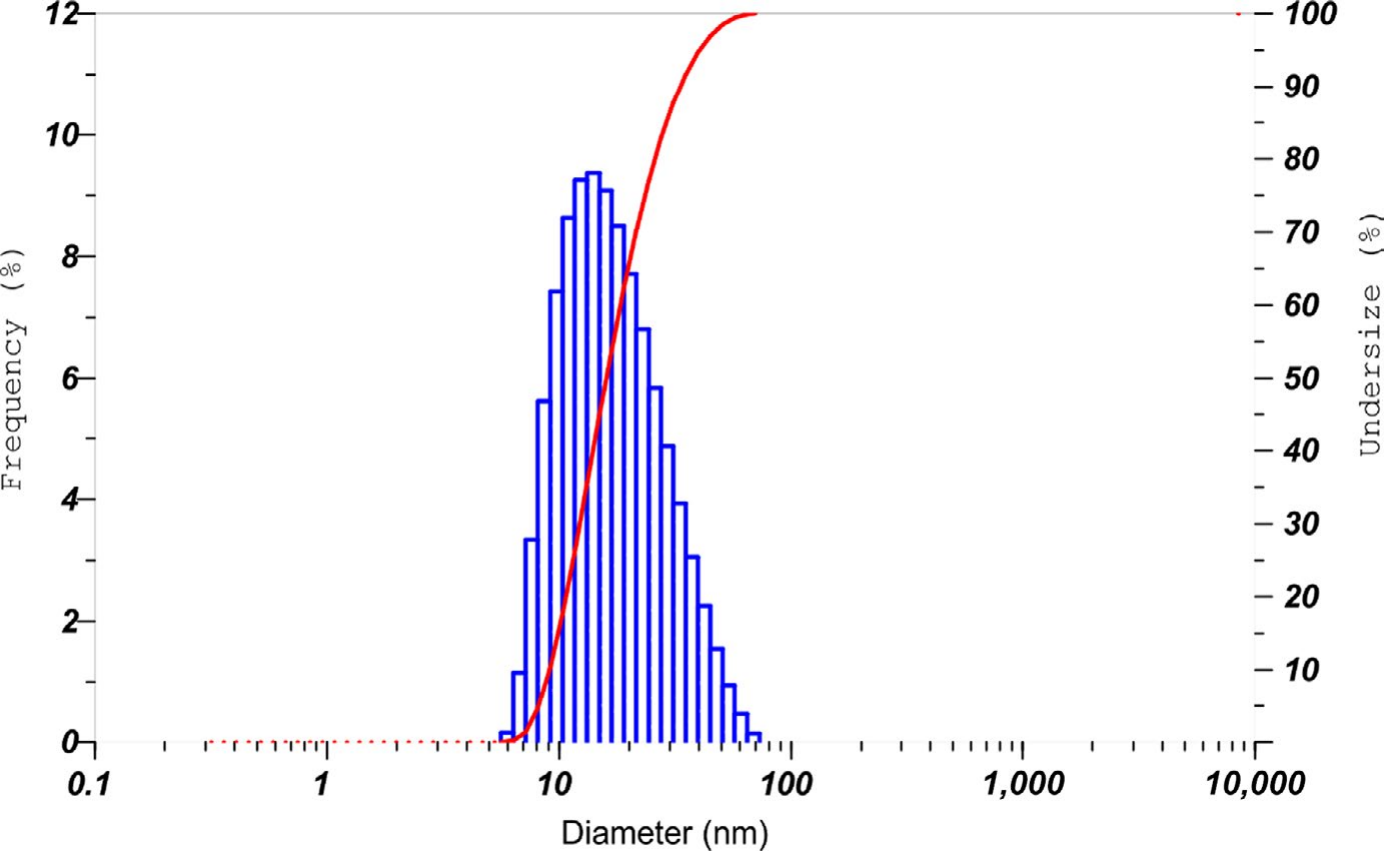
Diameter distribution of the nano‐emulsion with SOR 2.5 and heating temperature at 75°C.

Our reported results were also similar with a recent study where nano‐emulsion was fabricated with cinnamon oil (Chuesiang, Siripatrawan, Sanguandeekul, McClements, & McLandsborough, 2019). To be specific, as the SOR increased from 1 to 2, the average mean diameter of the cinnamon oil emulsion decreased from 100.76 to 23.52 nm. These results imply that the SOR is capable of determining the droplet diameter and the SOR of 2 could produce the emulsion with mean diameter of lower than 50 nm (Figure 10).

### 3.3 Construction of BPEO phase diagram and its evaluation

**Figure 11:**
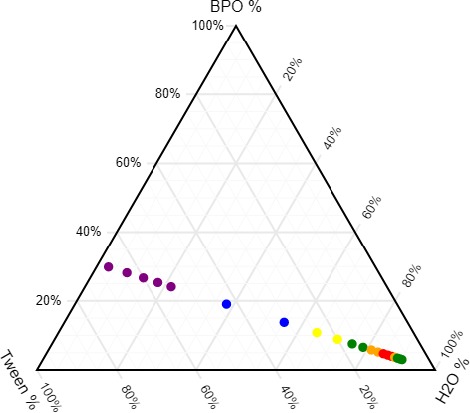
Phase diagram of the nano‐emulsion with SOR 2.25 and heating temperature at 75°C

Phase diagram of the nano‐emulsion with SOR 2.25 and heating temperature at 75°C was constructed by selecting BPEO as oil phase, Tween‐80 as surfactant and water as aqueous phase. It was constructed by performing aqueous titration according to Table 5. In the above phase diagram, large emulsion region (purple, orange, and green color) was obtained as compared to small nanoemulsification region. Gel region was obtained with aqueous/oil ratio from 2:1 to 8:1 (blue and yellow color). When the ratio of aqueous/oil was from 6:1 to 12:1 or from >24:1 two‐phase system was observed (yellow and green color). There was small nanoemulsification region, that is, single phase isotropic system observed toward aqueous rich apex, when aqueous/oil ratios were more than 18 and less than 22 (red color) as shown in Figure 11.

**Table 5:** Constituents found in black pepper essential oil and nano‐emulsion fabricated by PIT method

BPEO (ml)	Tween (ml)	H2O (ml)	Total (ml)	BPEO (%)	Tween‐80 (%)	H_2_O (%)	System state	Phase Numbers
1	2.25	0.1	3.35	29.85	67.16	2.99	Emulsion	1 (W/O)
1	2.25	0.3	3.55	28.17	63.38	8.45	Emulsion	1 (W/O)
1	2.25	0.5	3.75	26.67	60.00	13.33	Emulsion	1 (W/O)
1	2.25	0.7	3.95	25.32	56.96	17.72	Emulsion	1 (W/O)
1	2.25	0.9	4.15	24.1	54.22	21.69	Emulsion	1 (W/O)
1	2.25	2	5.25	19.05	42.86	38.10	Gel	1
1	2.25	4	7.25	13.79	31.03	55.17	Gel	1
1	2.25	6	9.25	10.81	24.32	64.86	Gel + Emulsion	2
1	2.25	8	11.25	8.89	20.00	71.11	Gel + Emulsion	2
1	2.25	10	13.25	7.55	16.98	75.47	Emulsion	2 (O/W)
1	2.25	12	15.25	6.56	14.75	78.69	Emulsion	2 (O/W)
1	2.25	14	17.25	5.80	13.04	81.16	Emulsion	1 (O/W)
1	2.25	16	19.25	5.19	11.69	83.12	Emulsion	1 (O/W)
1	2.25	18	21.25	4.71	10.59	84.71	Nano‐emulsion	1 (O/W)
1	2.25	20	23.25	4.30	9.68	86.02	Nanoemulsion	1 (O/W)
1	2.25	22	25.25	3.96	8.91	87.13	Nanoemulsion	1 (O/W)
1	2.25	24	27.25	3.67	8.26	88.07	Emulsion	1 (O/W)
1	2.25	26	29.25	3.42	7.69	88.89	Emulsion	2 (O/W)
1	2.25	28	31.25	3.20	7.20	89.60	Emulsion	2 (O/W)
1	2.25	30	33.25	3.01	6.77	90.23	Emulsion	2 (O/W)

### 3.4 Compositional determination of black pepper (*Piper nigrum* L.) essential oil by GC–MS

Volatile components of the essential oil were determined by GC – MS method with the Agilent 6,890 Plus (HP – USA) instrument. As shown in Table 6, the major components were 3‐carene (25.6%), followed by limonene (21.6%), β‐caryophyllene (20.7%), β‐pinene (12.6%), and α‐pinene (6.8%). These figures are similar to a previous study involving Vietnamese black pepper essential oil where 3‐carene was reported to be one of the major constituents (29.21%) (Tran et al., 2019). However, presented results were different from compositional results of essential oils of black pepper harvested from other areas where caryophyllene (about 10%–20%) and limonene (about 10%) were the major compounds (Feng, Jiang, Wang, & Li, 2010; Han, Beaumont, Rodriguez, & Bahr, 2018; Kumoro, Hasan, & Singh, 2010; Morshed, 2017; Teneva et al., 2016).

**Table 6:** Constituents found in black pepper essential oil and nano‐emulsion fabricated by PIT method

Constituents	Percent (%)	
	Essential oil	Nano‐emulsions
1. α‐Pinene	6.8	5.5
2. β‐Pinene	12.6	12.6
3. β‐Myrcene	2.6	2.6
4. 3‐Carene	25.6	10.4
5. α‐Phellandrene	4.4	–
6. Cymol	0.8	–
7. Limonene	21.6	8.35
8. Linalool	0.5	–
9. δ‐Elemene	1.1	–
10. α‐Copaene	2	2
11. α‐Caryophyllene	0.7	2.34
12. β‐Caryophyllene	20.7	13.24
13. δ‐Cadinene	0.5	–

**Figure 12:**
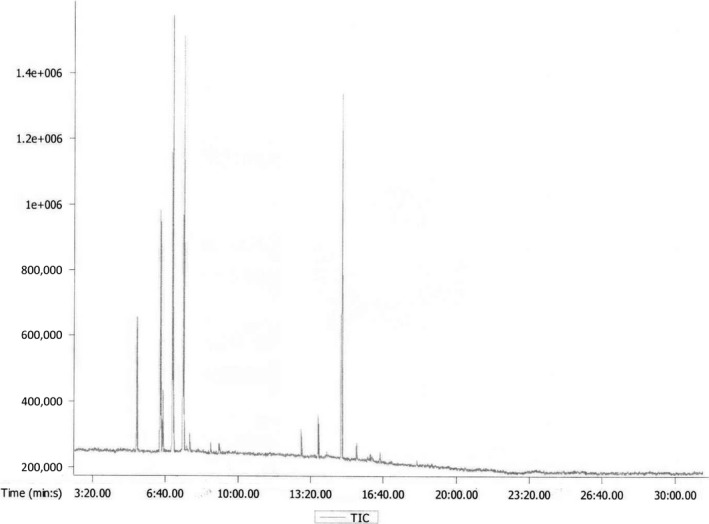
GC–MS spectra of the black pepper oil

**Figure 13:**
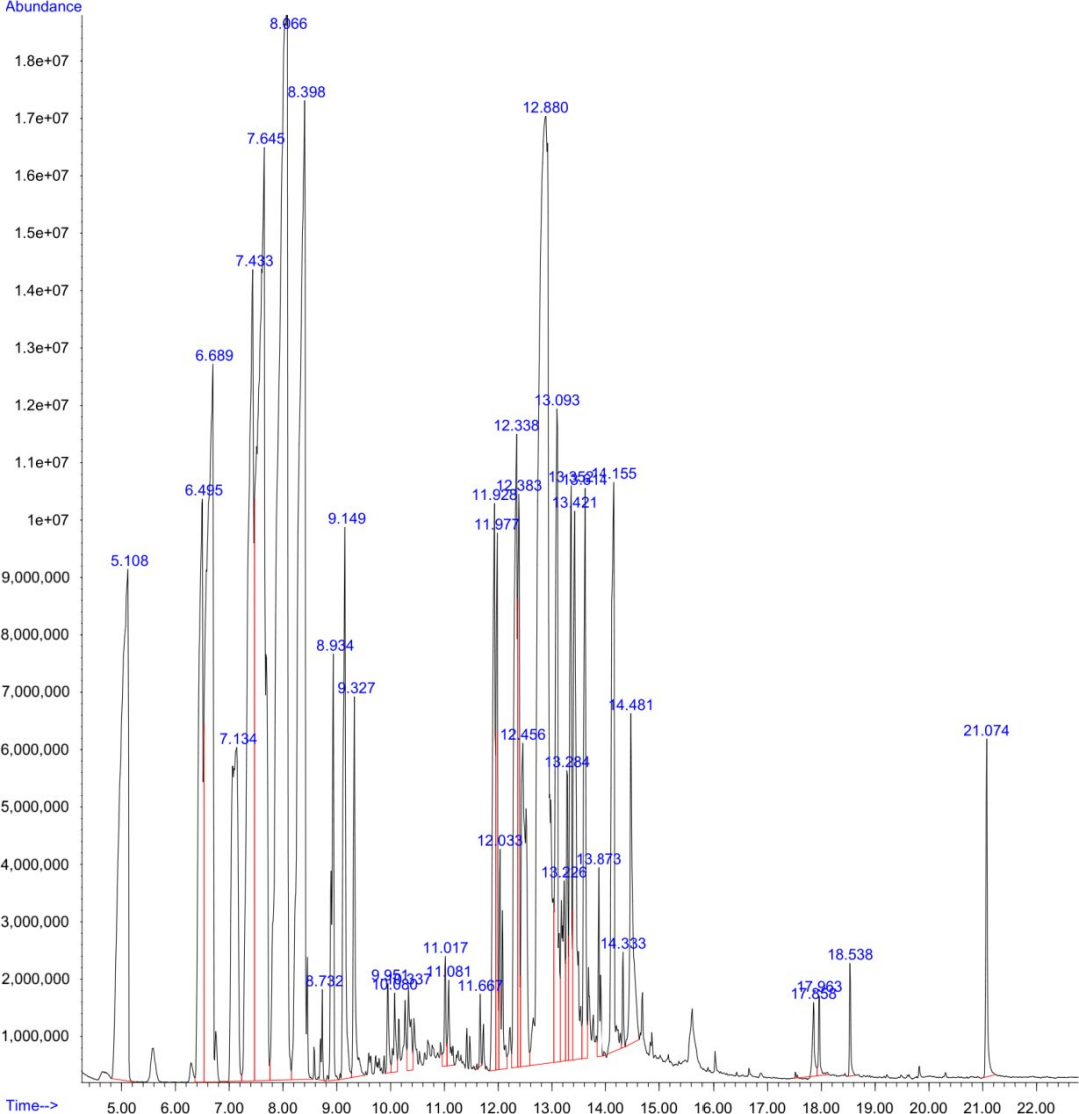
GC–MS spectra of the fabricated black pepper oil nano‐emulsion (B1)

After the nano‐emulsion had been successfully fabricated, another GC‐MS routine was performed to determine the composition of volatile components in the product as shown in Figure 12. Apparently, several constituents that were present in the bare essential oil were missing in the nano‐emulsion. Conversely, compared with the essential oil, the composition of the nano‐emulsion also exhibited a higher number of detected compounds (30 vs. 13). In addition, improved contents of α‐ and β‐caryophyllene were observed, which is indicative of the reaction and transformation of compounds in the oil into different compounds as shown in Figure 13. However, the presence and high concentration of important compounds (pipine, carene, limonene, and caryophyllene) were maintained after emulsification.

These compounds suggest that the fabricated nano‐emulsion exerts high antimicrobial activities. In a similar study, Bedoya‐Serna et al. (2018) utilized the PIT method to obtain the oregano essential oil nano‐emulsion and showed that nano‐encapsulated oregano essential oil presented an inhibitory effect against the three genera of fungi (*Cladosporium* sp., *Fusarium* sp., and *Penicillium* sp.). In another PIT attempt involving cinnamon oil, the formulated nano‐emulsion also exhibited inhibitory activities against a number of foodborne pathogens including *Escherichia coli*, *Salmonella typhimurium*, *Staphylococcus aureus,* and *Vibrio parahaemolyticus* thanks to the presence of cinnamaldehyde, which was retained in high concentration after emulsification (Chuesiang, Siripatrawan, Sanguandeekul, Yang, et al., 2019).

## 4 CONCLUSIONS

A black pepper essential oil nano‐emulsion system was successfully formulated by phase inversion temperature method, a facile and inexpensive technique. Examination of different experimental parameters revealed that the emulsification process that gave the homogeneous, smallest droplets with low PdI should be carried out with the ratio between Tween‐80 and the oil phase of 2.25:1 under the heating temperature of 75°C. When fabricated under these parameters, dispersion phase droplets of the fabricated nano‐emulsion could maintain the mean size of 17.9 nm, low polydiversity index (0.137), and transparency (OD < 0.05) after one month.

## CONFLICTS OF INTEREST

The authors declare no conflicts of interest.

## ETHICAL APPROVAL

This study does not involve any human or animal testing.
